# The impact of the educational marriage-matching model on the household income gap

**DOI:** 10.3389/fpsyg.2022.1082970

**Published:** 2023-01-06

**Authors:** Congjia Huo, Lingming Chen

**Affiliations:** ^1^Department of Economics, Business School (School of Quality Management and Standardization), Foshan University, Foshan, Guangdong, China; ^2^Department of Economics and Statistics, School of Economics and Management, Xinyu University (XYU), Xinyu, Jiangxi, China

**Keywords:** income gap, household income gap, educational homogeneity marriage, marriage-matching, Recentralization Influence Function (RIF), propensity score matching (PSM)

## Abstract

Education has become one of the important selection criteria for homogeneous marriage. The proportion of educational homogeneous marriage in China's marriage market is increasing. The inequality of family income is closely related to the educational background of family members. The article is based on the Chinese General Social Survey (CGSS) data. It uses the Recentralized Impact Function Regression Method (RIF) to empirically test that the marriage structure under different educational backgrounds has widened the income gap in Chinese households. Propensity score matching was used to correct possible selection bias and estimate the net effect of marital education matching on household income gaps. The results showed that the increase in the proportion of educational homogeneity in marriage would expand the family income gap. Based on theory and empirical evidence, some reasonable suggestions are put forward to advocate diversified marriages, strengthen social security, and reduce the family income gap.

## 1. Introduction

A widening income gap has accompanied China's rapid economic growth. The long-term change in the income gap can be divided into two stages. The first stage is the first 30 years of economic transformation. Academia agrees that the Gini coefficient is constantly rising in China. The second stage is 10 years after the economic change. There is still some academic disagreement about whether China's Gini coefficient is rising or falling. However, most scholars believe China's Gini coefficient has also been rising this decade. In 2018, the official Gini coefficient of the Chinese government was 0.474, far exceeding the international warning line of 0.4. The Gini coefficient of the net property of Chinese households once exceeded 0.5. In addition, high-income groups still have hidden income, and the data of the Gini coefficient may still be underestimated. At the same time, the income gap between urban and rural areas and regions is also widening.

**Figure 1:**
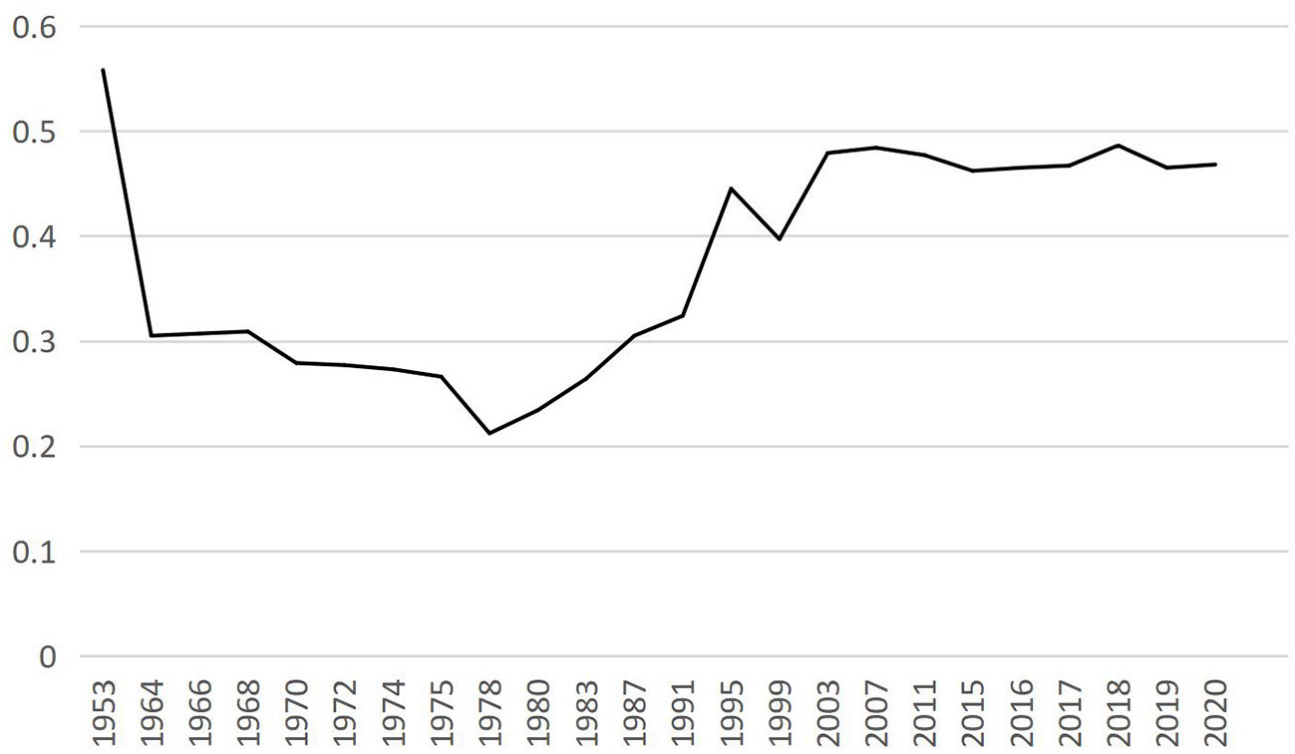
Gini coefficient of Chinese resident income from 1953 to 2020. The Gini coefficient of 2003–2020 is from the China yearbook of household survey (calendar years), the Gini coefficient of 1953–1978 is from the World Income Inequality Database (WIID), and the author estimates other years.

Figure 1 shows the Gini coefficient of Chinese residents' income from 1953 to 2020. From Figure 1, the Gini coefficient of residents' income in the early days of the founding of New China is exceptionally high. China's income gap has been narrowing with the establishment of a new economic system and the disappearance of the bureaucratic landlord class. Although the National Bureau of Statistics did not release the Gini coefficient of income before 2013, according to estimates, the Gini coefficient of early China's income gap was only about 0.3 (Adelman and Sunding, 1987). Then, the Gini coefficient of China's individual income rose steadily, reaching 0.468 by 2020. Personal income inequality is the basis of household income inequality. China is more family oriented. Generally speaking, income and expenditure are based on household units. Therefore, studying the household income gap can better reflect the status quo of income inequality in China. Marriage is the primary path that connects individuals and families. Different marriage matching patterns build other families, and families will affect the career and development of individuals, thereby further affecting family income.

Marriage has always been an important area of demographic and sociological research. As a basic social setting, marriage matching changes also reflect societal changes. The marriage between men and women is not a random combination but a comprehensive choice under specific social circumstances and social institutions. They must consider many factors, including ascribed factors (race, family conditions, parents' occupation, household registration, etc.) and self-contained factors (education background, occupation, age, appearance, personality, etc.). Generally, people will choose people with the same or similar characteristics as themselves as marriage partners.

China's social progress and economic development are also changing the pattern of marriage-matching. After the reform and opening up, China's politics, economy, and culture have undergone earth-shaking changes. Rapid economic development has laid a solid financial foundation for the freedom of marriage. Regarding the legal system, the “Marriage Law of the People's Republic of China” protects “freedom of marriage.” The law states that no one may interfere with the choice of the parties to a marriage, which has primarily weakened the family's stranglehold on young people's weddings.

On the other hand, improving the national education level has shaken the traditional Chinese concept of being a householder. It has also provided opportunities for men and women of the right age to contact each other. At the same time, the family planning policy has dramatically increased the proportion of only children, which has changed the inherent backward idea of “prioritizing sons,” and marriage is no longer a tool for family advancement. The popularization of higher education has been strengthened by the nine-year compulsory education system and the policy of expanding enrollment in colleges and universities. Women's educational attainment has also increased significantly, and the gender gap in education has narrowed. These provide the ground for educating homogenous marriages (marriages in which both parties have equal or the same level of education). The proportion of educationally homogenous marriages in China has continued to rise, and the combination marriage pattern of high-high education and low-low education has increased significantly.

The economic and social transformation is reshaping marriage patterns in China, and the change in educational marriage patterns will also impact the social structure and economic development. So, how does the increase in the proportion of educationally homogenous marriages affect the household income gap? Is it widening the income gap or narrowing it? In the current research, the measurement and decomposition methods of the income gap have formed a relatively complete and rigorous research system. Some scholars have researched family income inequality. Still, most relevant studies have discussed the family income gap from the perspective of intergenerational mobility. It ignores the impact of marriage matching on family heterogeneity. It lacks research on the effects of marriage education matching on the family income gap from the perspective of educational homogeneity. Based on the above background, this paper takes education homogeneity marriage and family income gap as the research object and estimates the impact of the educationally positive choice of marriage on family income inequality. The main innovation of this paper is to start with the marriage matching model, take the marriage education matching as the research object, explore the influencing factors of family income inequality, and also use the propensity score matching method to correct the possible selective bias, avoid the self-selection problem of marriage matching, eliminate the difference between the selected and unselected education homogeneous mating families through matching, and estimate the net impact of marriage education matching on family income gap, It provides new enlightenment for the study of alleviating the solidification of social strata and narrowing the family income gap. The second section of this article is a literature review. The third part gives the theoretical basis of marriage-matching. The fourth section examines the effect of positive educational marriage matching on the per capita household income gap through the Recentralized Impact Function (RIF) regression method. The fifth section uses the propensity score matching method (PSM) to verify the net effect of marriage education matching on the household income gap. The sixth section puts forward some policy recommendations based on empirical conclusions.

## 2. Literature review

The criteria for positive marriage selection matches include family background, region, age, social status, occupation, education level, income, and so on. In these criteria, people marry someone with similar characteristics due to their personal feelings or values, interests and hobbies, financial security, and other needs. With the development of the economy and social progress, factors such as occupation, educational attainment, and income play an increasingly important role in marriage-matching. The factors influencing marriage matching have changed from attribution to self-induced factors (Zijdeman and Maas, 2010; Zhu, 2017). Personal ability becomes the most critical bargaining chip in the marriage market. Among the self-induced factors, education level is one of the most influential factors. Educational attainment becomes an essential positive selection matching criterion in marriage-matching. In the marriage matching pattern, young men and women choose individuals with the same or similar educational level as their spouses, called educationally homogeneous marriage-matching. Marriages in which both parties have equal or the same academic level are called educational homogeneity marriages. Marriage in which members of society with different educational backgrounds get married is called educational heterogeneity marriage. With the popularization of 9-year compulsory education and the one-child policy, gender differences in education have gradually disappeared. At the same time, the gap between the education rates of males and females has steadily narrowed. At the same time, the employment rate of women has increased significantly, which is a prerequisite for an increase in educationally homogeneous marriage matches. From the 1970's to the present, the percentage of Educational Homogamy (EH) marriages has increased rapidly. The educational matching of husband and wife has a decisive influence on family, society, and individual development.

In Smits et al. (1998) study of educationally homogeneous marriage, data from 55 countries showed that young people are more likely to marry with educational homogeneity than older people. People with higher education attach more importance to the education level of their spouses. In comparison, people with lower education have a smaller range of spouse choices than people with higher education. It should be noted that modern society generally assumes that a member's educational level represents their social status (Blau and Schwartz, 2018). Higher education often corresponds to higher income in the labor market. Based on the theory of resource replacement in marriage, young men and women will also choose the person with the highest educational background as their spouse for the sake of offspring development and family climbing up the ladder (Hout, 1982; Macrae et al., 2018). Niu (2016) used data from surveys on the social status of Chinese women from 1990 to 2010 for his research. The study's results showed that the education level of both spouses was strongly associated with forming a consistent view of the marital relationship. Zhang (2003) found the persistence of class endogamy in China by studying the occupation and education level of the couple.

Marriage matching pattern certainly has a certain degree of influence on household income. Some studies have shown that education and homogenous marriage are significant drivers of widening the gap between the rich and the poor, bringing about polarization (Bernasco et al., 1998). At the same time, the inequality effect of family income brought about by educational homogeneity marriage matching is transitive from generation to generation (Song and Zhou, 2019). Greenwood et al. (2014) believed that Positive Assortative Matching (PAM) is a significant factor leading to income inequality. Using a dynamic model of marriage matching, Kremer (1997) found that the proportion of educated homogenous marriages in the United States increased in the 20th century without changing the income gap in the United States. There are many domestic and international studies on the effect of educationally homogeneous marital structure on the household income gap. The special status of education in the labor market determines its inevitable connection with income. Generally, the income effect of homogenous educational marriage can be studied from two perspectives.

### 2.1. Spillover effect

The spillover effect of education means that the education experienced by members of society will directly or indirectly affect their spouse's job, knowledge level, education, etc. (Birch and Miller, 2006). Generally speaking, a person's higher educational attainment can affect a spouse's educational attainment or occupation (Bernasco et al., 1998). Sociologists explain the special effects of homogeneous educational marriage from the perspective of the social communication network. It can be attributed to the externality of education. Benham (1974) research proves that a wife's education can affect her husband's income, increasing his annual income by about 3.5 percent. The cross-productivity theory of marriage suggests that wives can help their husbands improve their income through the information and skills they possess. In a combination family of low education and high education, men can increase their working hours through the division of labor at home (Becker, 1974). After marriage, women bear most of the housework, while men devote more energy to the workplace, increasing their income. However, the increase in such income is relatively low. The wife and husband have the same family combination with high education, and the spouse can increase each other's income by sharing resources, thus increasing the total family income.

### 2.2. Selection effect

The selection effect does not consider the increase in income of social members after marriage and only studies social members with high education and high income tend to choose spouses with the same education. It leads to a higher total family income after marriage among people with high academic qualifications, thus opening a gap with the whole family income of people with low educational qualifications. In other words, the increase in the household income gap results from educationally homogenous marriage choices. Dribe and Nystedt (2013) used Swiss household data to compare marital household income a decade before and after. The results show that households with higher-educated have higher total household incomes, and households with lower-educated have lower payments. That is: Educational homogeneity does not change household income. However, the revenue of family members with high educational uniformity is generally higher. The selection effect is more in line with the resource replacement theory in the marriage matching theory. Likewise, highly educated members of society can acquire traits in each other that enhance their knowledge and abilities. Apart from the emotional element of marriage, this point can better show the essence of human beings seeking advantages and avoiding disadvantages. Marriage is about getting a better life, with no difference between men and women.

Many scholars in China study the effect of educational marriage structure on the household income gap. Using CHIP data, Pan et al. (2015) found that the proportion of homogeneous educational marriages in China continued to increase between 1998 and 2008. The results show that the overall household income gap under the positive marriage model is more significant than the random matching family income gap. The role of educationally homogeneous marriage in expanding the family income gap is increasing yearly. Li et al. (2016) divided education matching into “upward matching,” “downward matching,” and “homogeneous matching.” The study found that women's choice of upward and homogeneous marriage and men's choice of upward marriage positively affected income. It shows that education has spillover effects.

## 3. Theoretical foundation

Since modernization, marriage has been brought into economics, and family economics has gradually linked marriage with the economy. Becker (1981) put forward the marriage matching theory in Discussion I and Discussion II of Family Economics, which became the basis for subsequent scholars to study marriage matching from an economic perspective. Specifically, the marriage matching theory sets up the marriage u and labor markets. In the market, social members abide by market behavior and assume that there are male social members *F* and female social members *M* in the marriage market. Only when the utility of both increases will they get married. The utility of both spouses in the marriage market depends on the goods “produced” by the family. In the marriage market, when rational people face many marriage candidates, looking for a combination object of choice is bound to maximize their interests. At the same time, this person is also a candidate for other rational people, so it can be assumed that there are n numbers of *F* and n numbers of *M*. In the random combination of *F* and *M*, there must be one or more combinations to obtain the maximum value of family products and social welfare.

This paper uses *Z*_*ij*_ to represent the maximum output of family products, *F*_*ij*_ is the family output when the *i* − *th* male chooses to maximize family output and the *j* − *th* female marries, and *M*_*ij*_ is the family output when the *i* − *th* female chooses to maximize family output and the *j* − *th* female marries, so the total family output is *Z*_*ij*_ = *F*_*ij*_ + *M*_*ij*_, and the total family output combination is shown in Formula (1).


(1) $$\begin{array}{l} Z_{ij}=|\begin{array}{cccc} Z_{11} & Z_{12} & \cdots & Z_{1n}\\ Z_{21} & Z_{22} & \cdots & Z_{2n}\\ \vdots & \vdots & \ddots & \vdots \\ Z_{n1} & Z_{n2} & \cdots & Z_{nn} \end{array}| \end{array}$$

The output of the marriage combination is different from that of the labor market. It is not measured by money or materials but by the quality of marriage life, emotional life, the number of children, etc. A certain number of unmarried people and social members cannot find a spouse and are forced to remain single. If they are not taken into account, then n members can have *n*+1 choice of the spouse, so it can be inferred that 2*n* social members have *n*^2^ + 2*n* choices. Therefore, the total output of all marriages in any combination is shown in Formula (2).


(2) $$\begin{array}{l} Z_{K}=\underset{i\in M,J\in F}{\sum }Z_{ij},k=1,2,3\cdots n \end{array}$$

It can be seen from Equation (1) that the maximum total output of marriage matching is on the diagonal of the matrix, as shown in Equation (3).


(3) $$\begin{array}{l} Z^{*}=\overset{n}{\underset{i=1}{\sum }}Z_{ij}=\max Z^{k} \end{array}$$

It can be seen from Formula (3) that when every member of society chooses a spouse who can let him obtain the maximum family output, society can also achieve the maximum utility combination. At this time, society reaches Pareto optimality, and no marriage matching mode increases the utility of one social member but does not decrease the utility of others. The diagonal effect is the same type of marriage matching. Sociology and economics have reached an agreement on this point–when a social member chooses to marry a social member with the same characteristics as himself, and the social utility can reach Pareto optimum. But for individuals, the Pareto optimality of society is not necessarily the best choice of individuals. For a specific social member, upward marriage is the best solution. However, every social member wants to get an upward marriage, and the final result is that most social members choose the same type of marriage. Marriage matching patterns can be divided into homotype and hetero-type matching. When two social members *F* and *M* have the same or similar characteristics in family background, income level, education background, age, race, etc., such marriage is homotype marriage. When the education level of both spouses is the same, it is educational homogeneity marriage. The input function of male *F* in family production is *A*_*f*_, and that of female *M* in family production is *A*_*m*_. When other conditions of two social members are the same, only the production input function is different, assuming that each feature in the production function increases monotonously in family output. The transformation function is shown in Formula (4).


(4) $$\begin{array}{l} \frac{\partial Z_{ij}\left(A_{m},A_{f}\right)}{\partial A}>0 \end{array}$$

The second derivative of equation (4) is: $\frac{\partial Z\left(A_{m},A_{f}\right)}{\partial A_{m},\partial A_{f}}$ When *A*_*m*_ and *A*_*f*_ are positively correlated, the second derivative of the family total output function is more significant than zero, and the total output can reach the maximum. When *A*_*m*_ and *A*_*f*_ are negatively correlated, the second derivative of the family total output function is more significant than zero, and the total output is the minimum. Therefore, when the characteristics of both husband and wife are the same, their output is positively correlated, and the marriage matching pattern can achieve the best effect at this time. When both spouses have the same religion, education, family background, income level and other characteristics, the same concepts and living habits can increase happiness after marriage. Of course, complementary marriages can also improve the quality of marital life, but in this way, both husband and wife need to maintain stronger feelings.

Becker's marriage theory summarized the characteristics such as age, educational background, color, religious belief and emotional experience as positive matching factors of marriage and the replaceable characteristics such as income, family background and material base as negative matching factors. If both spouses in the family have high incomes, such a combination will cause a waste of resources to a certain extent. Because the family is an economical combination unit based on the division of labor, the time of two spouses must be allocated to the maintenance of the family. The income level of everyone in the family is high, which will naturally reduce the time for family production and maintenance. From the perspective of utility, it will reduce the total utility of the family, which is not conducive to the stability of the family. The division of labor in the family makes the marriage-matching features complementary to some extent. At this time, the optimal utility of the family is shown in Formula (5).


(5) $$\begin{array}{l} U=\frac{W}{C\left(s_{m},s_{f},p,A_{m},A_{f}\right)} \end{array}$$

In Formula (5), the input function of male *F* in family production is *A*_*f*_, and that of female *M* in family production is *A*_*m*_. *P* is the rate of return on non-human capital. Assuming that the input function of both spouses in production is independent of marriage matching factors, the numerator and denominator in Formula 4–5 are derived from *A*_*m*_ and *A*_*m*_ respectively, so that their derivatives are more significant than 0. After simplification, Formula (6) and (7) is obtained.


(6) $$\begin{array}{l} \frac{\partial Z}{\partial A_{m}}=-WC^{-2}C_{m}>0 \end{array}$$

(7) $$\begin{array}{l} \frac{\partial Z}{\partial A_{f}}=-WC^{-2}C_{f}>0 \end{array}$$

Then, the second derivative of Equations (6), (7) is calculated to verify that the second derivative is positive. It also proves that if men's investment in family production and women's investment in family production is independent, the marriage mode could achieve the best family production only when the positive mating factors complement each other.

Becker's marriage theory oversimplifies the purpose and significance of marriage. Based on Becker's marriage theory, some scholars put forward the marriage search theory. They believe that “marriage matching is based on marriage wages, that is, the share of marriage output expected by the pursuer after marriage with his potential spouse, to measure whether the potential spouse is suitable” (Boulier and Rosenzweig, 1984; Fernandez et al., 2005). However, in reality, there is a great degree of lack of information about potential spouses in the process of marriage search. Individuals cannot directly observe marriage wages when looking for spouses. Therefore, individuals need to spend time, energy and money to judge the distribution of marriage wages to predict the expected output of potential spouses' marriage and make decisions. This search process is also random. When two people judge that the expected output of each other after marriage is greater than the cost of continuing their search, they will choose to become spouses. Looking for the characteristics of potential spouses in the marriage theory has become the basis for predicting the expected effect of marriage. With economic development, it is customary in modern society to divide a person's social status and economic capacity by the degree of schooling (Blau and Schwartz, 2018). The education level of potential spouses has become one of the essential characteristics for estimating marriage wages.

## 4. Empirical research

### 4.1. Data sources

The data used in this article is from the China Household Income Survey (CHIP), which is the data from 1995, 1999, 2002, 2007, 2008, and 2013. The survey cycle of CHIP data is 5 years. The most recent survey was conducted in 2018, but this data is not published temporarily. The change of marriage matching mode is relatively slow, so the survey data in 2013 is still representative. The CHIP survey has many missing values in the rural household survey and the floating population survey, and the information is imperfect. Therefore, this paper only uses the data from the urban household survey. This article uses the data from the China household income survey in 2013. The data only retained the sample data of first marriage and remarriage, excluding unmarried, cohabitation, and widowed samples. Then, both spouses' sample data of household income and the lack of education were removed. According to the legal marriage age in China, only the sample data of men aged 22 and above and women aged 20 and above were retained, and finally, 5,455 sample data were obtained.

### 4.2. Model construction and variable setting

Unlike the traditional least squares regression method, the explained variable in the re-centered influence function (RIF) regression method can be either the income level or the quantile and variance obtained based on the influence function. Therefore, this paper verifies the impact of education on homogeneous marriage on family income inequality from the distribution perspective. In addition, the RIF method can effectively weaken the endogenous problem caused by omitted variables, and the empirical results are more robust.

The Gini coefficient (Gini, 1912) is the most commonly used indicator to measure inequality. It has a more intuitive economic meaning and is not affected by the sample size. The Gini coefficient is the most widely used index for measuring the income gap. The curve fitting method has strong operability and applicability in calculating the Gini coefficient. According to different curve equations used for fitting, it can be divided into polynomial function method, generalized quadratic function method, etc. (Zhao, 2011). And the other is the calculation formula of the Gini coefficient in discrete form (Liu and Tian, 2009). Discrete distribution is only suitable for numerical data, but it can cover more survey information; Continuous distribution can be subdivided into parameter estimation and semi-parameter estimation, but the premise of parameter estimation is to determine a specific function form. Then we estimate the parameters through survey data to obtain the income distribution function (McDonald and Jensen, 1979).

This paper uses the Gini coefficient to measure household income inequality. The recentralized influence function is formulated as follows:


(8) $$\begin{array}{l} RIF\left(y;v^{Gini}\right)=1+2\mu^{-2}R\left(F_{y}\right)y-2\mu^{-1}\{y\left[1-p\left(y\right)\right]\\ +GL\left[p\left(y\right);F_{Y}\right]\} \end{array}$$

Where, *v*^*Gini*^ is the Gini coefficient corresponding to the income distribution *F*_*Y*_, μ is the expected total revenue, GL is the generalized Lorentzian curve, R is the integral of the generalized Lorentzian curve on [0, 1], p is the income distribution, and *F*_*Y*_ is the corresponding proportion of the cumulative population not higher than the income level y. To estimate the impact of education-positive marriage on household income inequality, the benchmark regression model of the RIF regression method was constructed as follows:


(9) $$\begin{array}{l} Gini\left(cinco\right)=\alpha edu\text{\_}mar+\beta X+\omega \end{array}$$

Where, *Gini*(*cinco*) is the Gini coefficient of per capita household income, which measures household income inequality. *edu*_*mar* is a dummy variable that measures whether it is an educationally homogeneous marriage. *X* is the control variable and ω is the random error term.

The concepts, setting, and assignment methods of the main variables in this paper are as follows:

Explained variable: per capita household income (*cinco*). Household per capita income is a better measure of household income levels. Per capita household income = total household income/total household population. The whole household income includes all kinds of labor remuneration for all family members, including wages, bonuses, subsidies, contract income, interest, dividends, pension income, income from running or owning individual or private enterprises, etc.

Explanatory variable: whether Education homogenous marriage or not (*edu*_*mar*). The education level of the respondents and their spouses is given in detail in the CHIP questionnaire. According to the specific national conditions in China, this paper divides academic qualifications into four levels according to the length of education, which are as follows:

① Education below senior high school (A-), including junior high school, primary school, literacy classes, and no schooling;
② High school education (B-), including high school, technical secondary school, and higher vocational education, which are equivalent to high school education;
③ Above senior high school and below university (C-), including junior college degree;
④ University degree and above (D-), university and graduate degree and above.

The spouse education gap refers to the difference between the husband's and wife's education. This paper does not strictly distinguish whether the husband's educational background is higher than the wife's or the wife's educational background is taller than her husband's and takes the absolute value as the statistical data. When spouses have the same educational experience, they are educationally homogeneous marriages, assigned a value of 1, and non-educationally homogeneous marriages are assigned a value of 0.

Control variables: The control variables are roughly divided into two aspects: household characteristics and family characteristics. Household head characteristics include the following:

① The householder's age (*age*);
② Number of years of schooling for the head of household (*edu*);
③ Head of household health level (*hea*): This is the subjective assessment of the head of household, divided into five grades: excellent, good, average, bad, and terrible. In this paper, the rating level was used as the dummy variable of the householder's health level;
④ Whether the head of the household has a job (*job*): The head of a family with a regular job is assigned 1, and the head of a family without a steady job is assigned 0;
⑤ Whether the head of the household is a party member (*par*): The head of the household is a member of the Communist Party of China assigned a value of 1, the head of the family is not a member of the Communist Party of China assigned a value of 0.
⑥ Number of husband and wife workers (*emp*): This variable is the number of couples with regular jobs, with both having regular jobs recorded as 2, with only one of the couples having a steady job recorded as 1, and with neither of the couples having a stable job recorded as 0.
⑦ Family size (*fam*): This variable represents the total number of people in the household.
⑧ Age the difference between husband and wife (*edu*_*age*): The calculation here is the absolute value of the age difference between spouses, without distinguishing between husband and wife.
⑨ Province of the family (*pro*): CHIP urban data is only available for 14 provinces surveyed, which are assigned to the provinces respectively. 1 Beijing municipal, 2 Shanxi Province, 2 Liaoning Province, 4 Jiangsu Province, 5 Anhui Province, 6 Shandong Province, 7 Henan Province, 8 Hubei Province, 9 Hunan Province, 10 Guangdong Province, 11 Chongqing Municipality, 12 Sichuan Province, 13 Yunnan Province, 14 Gansu Province. Table 1 shows the descriptive statistical analysis of variables.

**Table 1:** Descriptive statistical analysis.

**Variable**	**Mean**	**Standard deviation**	**Minimal value**	**Maximum value**
Per capita household income (*cinco*)	26,939.75	21,357.03	0	617,000
Whether education homogenous marriage (*edu*_*mar*)	0.6161	0.48638	0	1
The householder's age (*age*)	50	12.28	22	90
Number of years of schooling for the head of household (*edu*)	10.78	3.44	0	21
Head of household health level (*hea*)	2.1	0.876	1	5
Whether the head of the household has a job (*job*)	0.9266	0.2608	0	1
Whether the head of the household is a party member (*par*)	0.2934	0.4554	0	1
Number of husband and wife workers (*emp*)	1.2336	0.8438	0	2
Family size (*fam*)	3.1464	1.0597	2	9
The age difference between husband and wife (*age*_*gap*)	2.7655	3.157	0	47
Province of the family (*pro*)	6.7878	4.032	1	14

### 4.3. Evidence results

Table 2 shows the RIF regression results of the benchmark model of educational homogeneity and marital influence on household income. The explained variable is the Gini coefficient of per capita household income. Regression coefficients are the marginal effects of different variables on the Gini coefficient. In Table 2, model (1) only includes the critical variable of whether it is an educationally homogeneous marriage. A series of variables, such as householder characteristics, are gradually added to the model (2) and model (3). The family characteristics variable is added to the model (4). At the same time, models (1)–(4) all fix the province dummy variables. In model (5), the dummy variable of the province is not added for comparison.

**Table 2:** Baseline estimation results of the impact of educational homogeneity on the family income gap.

**Variable**	**Model (1)**	**Model (2)**	**Model (3)**	**Model (4)**	**Model (5)**
edu_mar	0.0094^***^ (3.89)	0.0329^***^ (3.48)	0.0319^***^ (3.08)	0.0323^***^ (3.02)	0.0326^***^ (2.95)
*age*		0.0021 (0.77)	0.0026 (0.90)	0.0025 (0.87)	0.0031 (1.08)
*age* 2		−0.00003 (−1.19)	−0.00003 (−1.29)	−0.00003 (−1.00)	−0.00003 (−1.20)
*par*		−0.032^***^ (−3.47)	−0.0294^***^ (−2.79)	−0.0287^***^ (−2.77)	−0.0329^***^ (−3.07)
*edu*			0.0017 (0.91)	0.0023 (1.23)	0.0036^*^ (1.84)
*hea*			−0.0108^*^ (−1.73)	−0.0093 (−1.52)	−0.0104^*^ (−1.67)
*job*			−0.1185^***^ (−6.61)	−0.125^***^ (−6.82)	−0.121^***^ (−6.55)
*emp*				0.0142^*^ (1.71)	0.0128 (1.60)
*fam*				0.0132^**^ (2.53)	0.014^***^ (2.62)
*age*_*gap*				0.0037 (1.35)	0.0042 (1.61)
Intercept item	0.32^***^ (48.59)	0.3073^***^ (4.42)	0.4028^***^ (4.80)	0.3124^***^ (3.25)	0.2787^***^ (2.92)
Provincial dummy variable	Yes	Yes	Yes	Yes	No
R^2^	0.0094	0.0128	0.0194	0.0221	0.0158
observations	5455	5455	5455	5455	5455

The t-value is in parentheses.

***, ** and * represent the 1, 5, and 10% significance levels, respectively.

Comparing model (1) to model (5), we can find that the estimated coefficients of educationally homogeneous marriage are all positive at the 1% significance level. It shows that the marriage pattern of educational homogeneity will indeed expand family income inequality. Homogeneous marriage in education allows young people with the same educational background to form families. Higher Education is more likely to bring higher income, making the rich richer and the poor poorer. On the other hand, highly educated couples prefer late marriage, late childbearing, and eugenics, and the family size is smaller. In the case of the same total household income, the per capita income of such households will be higher. The opposite is true for couples with low education. It has led to further widening of per capita household income inequality. Yue (2021) and Jiang (2018) reached the same conclusion as this article. Sun proposed that positive homogeneous families within the education matching dimension have the strongest effect on the improvement of family income level, followed by the superposition of double matching dimensions. The intra-group differences between homogeneous marriage families have a heterogeneous impact on family income levels. Xu and Cao (2022) calculated the Gini coefficient of household income at different levels of education. Then it is found that the Gini coefficient of urban and rural families after random matching is significantly lower than that of positive selection matching. Xu and Zhou (2020) demonstrated the same view from the perspective of household income of the floating population.

From a series of control variables in the model (4), the age and the education level of the head of the household have no significant effect on the household income gap. The head of household has a job, and whether they are a party member is significantly negative at 1%. It means that the head of the family having a job and the head of the household being a party member can effectively reduce the household income gap. In the series of control variables of household characteristics, the number of couples with jobs is significantly positive at the 10% level. In other words, the larger the number of working couples, the higher the total household income and the more significant the income gap. The age difference between husband and wife has no significant effect on family income inequality. The household size is significantly negative at 5%, and the increase in household size will widen the household income gap.

### 4.4. Robustness test

The robustness test examines the robustness of the evaluation method and indicator interpretation ability, that is, whether the evaluation method and indicator still maintain a relatively consistent and stable interpretation of the evaluation results when some parameters are changed. The RIF regression program operation uses standard robustness error by default, which can effectively weaken the endogenous problems caused by omitted variables etc., and avoid heteroscedasticity interfering with the estimation results. In addition, the per capita household income and the educational marriage patterns are not in the same year, so endogenous existence is less likely. To ensure the robustness of empirical research, this paper uses the replacement of the income inequality measurement index, the reclassification of education levels, the replacement of explained variables, and the change of explanatory variables to test the robustness of conclusions. Specific test results are shown in Table 3.

(1) Income inequality was measured using the 90–10th percentile. The quantile distance is more likely to examine the income gap between the highest and lowest income groups. In this paper, the 90–10 quantile distance is used to replace the Gini coefficient to test the robustness of the empirical results. The regression results are shown in model (6) of Table 3. The regression result was significantly positive at the 1% level, with the corresponding regression coefficient being 0.6034.
(2) Reclassify education levels. The classification standard of education attainment is essential for studying educational marriage. The previous section divides the degree of education into four groups. To test the robustness of the empirical results, this paper divides the education level into three levels. Specifically, it includes high school or below, high school or equivalent high school, and college degree or above. The assignment method of homogeneous educational marriage is the same, and the specific regression results are shown in model (7) in Table 3. The regression result is significantly positive at the level of 1%, and the coefficient in the corresponding regression is 0.0339. The results show that no matter how the level of education is divided, homogeneous education marriage will expand household income inequality.
(3) Replacement of explanatory variables. This paper divides education level into four groups, with values of 1–4. The spouse education gap is calculated by assignment, and the spouse education gap is used to replace the positive matching of education—the specific regression results in the model (8) of Table 3. The regression result is significantly negative at 1%, and the coefficient *edu*_*gap* in the corresponding regression is −0.0204. It shows that the smaller the educational gap between spouses, the higher the household income inequality. It also indicates that the benchmark regression results of this paper are robust.
(4) Replacement of explained variables. Compared with income, consumption expenditure can better reflect a household's income level. Moreover, consumption data is more authentic and reliable than income data, so this paper uses the Gini coefficient of per capita consumption of households to replace the Gini coefficient of per capita household income to test the robustness of the empirical results. The regression results are shown in model (9) in Table 3. The regression result is significantly positive at the level of 5%, and the coefficient of *edu*_*mar* in the corresponding regression is 0.0236. This value is not substantially different from the regression result of household per capita income as the explained variable in Table 2, which shows that the empirical result is robust.

**Table 3:** Robustness test results.

**Explanatory variables**	**90–10 Quantile moment**	**Three levels of education**	**Replacement of explanatory variables**	**Replacement of explanatory variables**
	**Model (6)**	**Model (7)**	**Model (8)**	**Model (9)**
*age*_*mar*	0.6034^***^ (2.98)	0.0339^***^ (3.35)	—	0.0236^**^ (2.04)
*age*_*gap*	—	—	−0.0204^***^ (−2.60)	—
Control variables	Yes	Yes	Yes	Yes
Intercept item	2.922^*^ (1.68)	0.3012^***^ (3.11)	0.3385^***^ (3.63)	0.3453^***^ (2.6)
Provincial dummy variable	Yes	Yes	Yes	Yes
Observations	5,455	5,455	5,455	5,455

The t-value is in parentheses.

***, ** and * represent the 1, 5, and 10% significance levels, respectively.

### 4.5. Analysis of influence mechanism

RIF regression results verify that educational marriage patterns affect household income inequality. Next, this paper demonstrates the pathways of the impact of the educational marriage mode.

**Figure 2:**
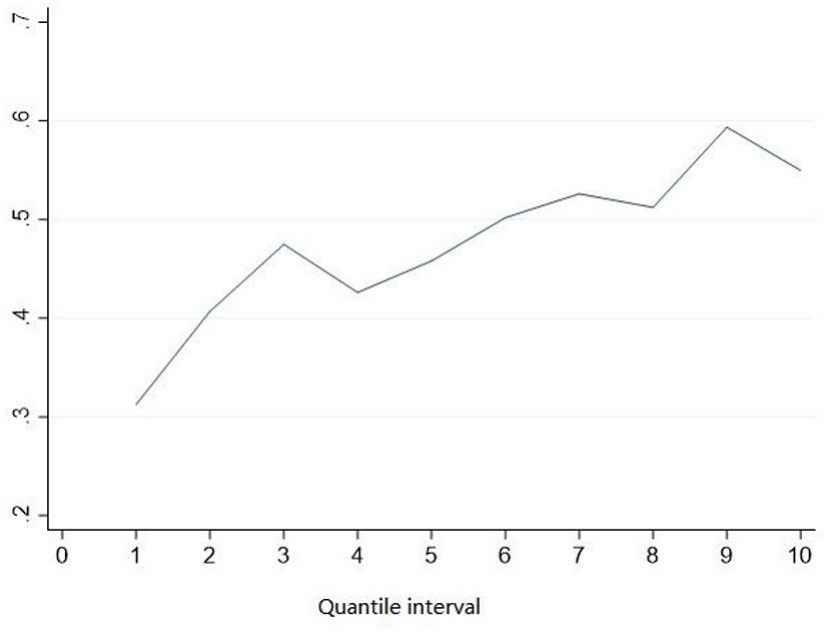
Means of positive education matching between household per capita income quartiles.

First, the income of human capital is directly proportional to the level of education. The return rate of education increases with the increase in education level. With the rapid expansion of higher education in China, the market continues to increase the demand for jobs for highly educated workers. People with low education are forced to take lower-paying jobs. Then the gap between the household income of highly-educated and low-educated couples is bound to grow, which is shown in Figure 2. This paper divides the samples into ten equal groups, from lowest to highest, according to per capita household income. Then calculate the mean value of the education positive marriage dummy variable (*edu*_*mar*) in each quantile interval. As seen from Figure 1, with the increase in income subgroups, the mean value of positive education matching is also increasing, showing a positive correlation. In the first quartile subgroup, this data is only about 0.3. In the sixth quartile group, this value has reached more than 0.5. These facts show that the rich rather than the poor are more likely to choose educationally homogeneous marriage. Families who decided on educationally homogeneous marriages had higher incomes and received a higher return on education.

**Figure 3:**
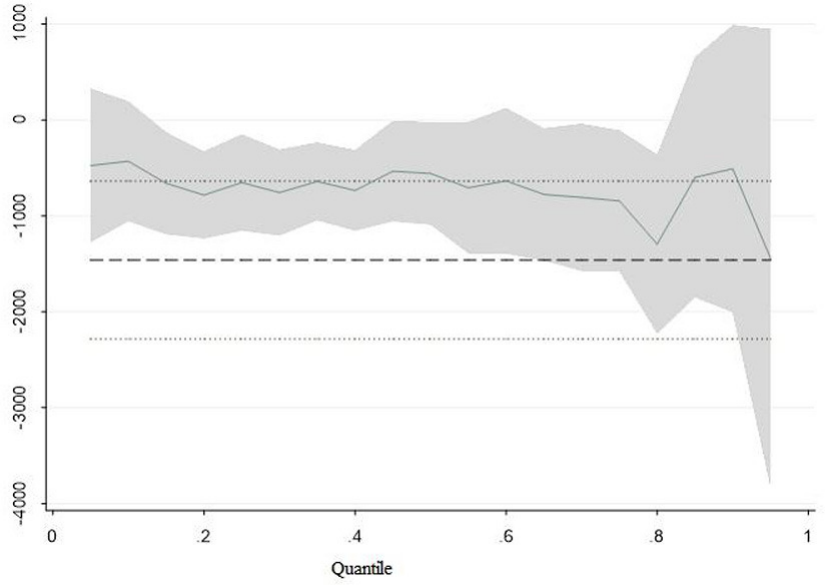
Marginal contribution rate of spouse education gap at different income quantiles.

Second, families with different levels of education have different abilities to accumulate wealth, which tends to produce a noticeable “Matthew effect.” It further exacerbates household income inequality. Figure 3 takes the household per capita income as the explained variable and the spouse education gap (*edu*_*gap*) as the core explanatory variable. Then add other control variables to show the change of the quantile regression coefficient with quantile variation more visually.

Figure 3 shows that the overall regression coefficient of the spouse education gap decreases as quantile changes. And this regression coefficient is significant at different quantiles. At both ends of the conditional distribution, the 95% confidence interval becomes wider. It shows that the spouse education gap does not equally affect families with different income levels. At the low quantile, the regression coefficient is even positive. It is because the household income of the combination of low Education and low Education is smaller than that of the combination of low education and high education. At the high quartile, this value was once lower than −1,000. It shows that compared with low-income families, high-income families are more likely to choose the homogeneous educational marriage mode and get a higher return on education. In low-education families, inadequate education of family members leads to low income for each member and a lack of sufficient resources to help spouse increase income. Moreover, such families do not have educational resources to improve the education level of their children, and intergenerational transmission will further widen the family income gap between low-low-education families and high-high-education families. On the whole, education homogeneous marriage further aggravates the inequality of income distribution and also promotes the widening of the household income gap through the “Matthew effect.”

## 5. PSM verifies the net effect of marriage-education matching on the household income gap

### 5.1. Method description

In the previous section of the paper, the Recentralization Influence Function (RIF) regression method is used to examine the effect of marriage education matching on the household income gap. Although the RIF method can effectively weaken the endogenous problems caused by omitted variables, there may also be the problem of self-selection of marriage matching in empirical analysis. In other words, the decision-making choices of marriageable men and women for their spouses are not strictly exogenous. Whether children choose family background, educational qualifications, income level, occupation, region, and others factors will also influence homogeneous education marriage. It may lead to the problem of self-selection bias, resulting in biased estimation results. Therefore, to identify the “net effect” of marriage education matching on household income inequality as much as possible, this paper uses the propensity score matching method (PSM) to correct the potential selective bias.

Propensity Score Matching is generally used to process data from observational studies. There are many biases and confounding variables in observation and research due to various reasons. The propensity score matching method reduces the influence of these deviations and confounding variables, to make a more appropriate comparison between the experimental and control groups. This method was first proposed by Rosenbaum and Rubin (1983). It can weaken the influence of hybrid variables between the experimental and control groups, making it easy to produce systematic deviation. Tendency score matching is used to solve this problem and eliminate interference factors between groups.

The propensity score matching method (PSM) does not directly compare the selection of educationally homogeneous marriage match groups with the non-educationally homogeneous marriage match group. After calculating the propensity score of marriageable men and women to choose homogenous educational marriage, PSM matched the most comparable families with educational homogenous marriage matches in multiple dimensions. It was used as a control group of educational homogeneous marriage family groups. Then by comparing the difference in relative income deprivation index between the two groups of households, we can get the net impact of marriage education matching on the household income gap.

CHIP data includes a wealth of personal, family, and community information. This paper uses the PSM method to verify the empirical results of the last part. The section uses the same CHIP data from 2013, and the processing method is the same as in the previous quarter. PSM made no significant difference between families who selected and did not select educationally homogeneous marriages by matching. Finally, it can effectively address the estimation bias caused by omitted variables and control the bias caused by the self-selection of marriage education matching. The specific calculation steps are as follows.

(1) Get propensity matching scores

The propensity score matching method first needs to calculate the propensity matching score. Rosenbaum defined “the propensity score as the conditional probability of an individual receiving processing after a given multidimensional eigenvector” (Rosenbaum and Rubin, 1983). Accordingly, the functional expression of the propensity score matching score is shown in Equation (10).


(10) $$\begin{array}{c} \alpha =P\left(X\right)=\mathrm{Pr}\left[edu\text{\_}mar=1|X\right]=E\left[edu\text{\_}mar|X\right] \end{array}$$

Equation (3) α is the score value of propensity matching, which is the probability of selecting educational homogeneity marriage match in the sample. *edu*_*mar*{0, 1} means whether it is in the treatment group or not. *edu*_*mar* = 1 is the treatment group, indicating families formed by homogeneous educational marriages. *edu*_*mar* = 0 is the control group, showing families formed by educational heterogeneity marriage. *X* is the factor that affects the marriage education match, which is the matching variable. In practice, it is generally impossible to measure the propensity score directly. Therefore, this article uses Dehejia's logit model to estimate the propensity score value of marriage education matching. The function expression is shown in Equation (11).


(11) $$\begin{array}{c} \alpha_{i}=P\left(edu\text{\_}mar=1|X_{i}\right)=\frac{exp\left(\nu X_{i}\right)}{1+exp\left(\nu X_{i}\right)} \end{array}$$

The premise of propensity score matching is to obtain an accurate propensity matching score. It means that *x* is evenly distributed between the matched treatment group and the control group. This process is called “data balancing.” Based on this, the balance test will be performed in the empirical study. The difference in measurement units leads to the gap between the control and treatment groups. To obtain the balance data, this paper first carries out the standardized deviation test for each *x* of *X*, as shown in Equation (12).


(12) $$\begin{array}{c} \frac{\left|{\bar{x}}_{treat}-{\bar{x}}_{control}\right|}{\sqrt{\left(s_{x,treat}^{2}+s_{x,control}^{2}\right)/2}} \end{array}$$

In Equation (5), $s_{x,treat}^{2}$ and $s_{control}^{2}$ are the sample variances of the variables of the treatment and control groups, respectively. Generally speaking, the standardization gap does not exceed 10%. The propensity score must be re-estimated when the standardized variance exceeds 10%.

(2) Perform propensity score matching

According to the matching principle, after the sample data pass the balance test, there is no significant difference in matching variables between families who choose education homogeneous marriage and families who decide education heterogeneous marriage. The only difference was whether to choose homogeneous educational marriage. Then calculate the average processing effect of the treatment and control groups for the matched samples. That is the net impact marriage education reaching on family income inequality. According to Becker and Ichino's mean treatment effect calculation method, the sample's functional expression of the mean treatment effect (ATT) estimator is shown in Equation (13).


(13) $$\begin{array}{c} ATT=\frac{1}{N_{1}}\sum_{i:edu\text{\_}mar_{i}=1}\left(y_{i}-{\overset{⌢}{y}}_{0i}\right) \end{array}$$

Equation (13) *N*_1_ = ∑_*i*_*edu*_*mar*_*i*_ is the treatment group, the sample size of educationally homogeneous married families. ∑_*i* : *edu*_*mar*_*j*_ = 1_ indicates that only the samples of the treatment group are aggregated. *Y* is the dependent variable and the relative deprivation index of income. *y*_1*i*_ and *y*_0*i*_ represent the relative deprivation index of household income in the same household under two scenarios: Spouses' choice of educationally homogeneous marriage and educationally heterogeneous marriage, respectively. Similarly, the corresponding match of the treatment group can be found for the control group, that is, the educationally heterogeneous marriage and family group sample *j*. The functional expression of the average treatment effect (ATE) estimator is Equation (14).


(14) $$\begin{array}{c} ATU=\frac{1}{N_{0}}\sum_{j:edu\text{\_}mar_{j}=0}\left({\hat{y}}_{1j}-y_{j}\right) \end{array}$$

Equation (14), *N*_0_ ∑_*j*_(1 − *edu*_*mar*_*j*_) is the control group, which is the number of samples of educationally heterogeneous marriages. ∑_*j* : *edu*_*mar*_*j*_ = 0_ indicates that only the control group samples were summed. The functional expression of the average treatment effect (ATE) estimator of the whole sample, including the control and treatment groups, is shown in Equation (15).


(15) $$\begin{array}{c} ATE=\frac{1}{N}\sum_{i=1}^{N}\left({\hat{y}}_{1i}-{\hat{y}}_{0i}\right) \end{array}$$

Equation (15) *N* = *N*_0_ + *N*_1_ is the sample size. If *edu*_*mar*_*i*_ = 1, then ${\hat{y}}_{1i}=y_{i}$; If *edu*_*mar*_*i*_ = 0, then ${\hat{y}}_{0i}=y_{i}$.

(3) Selection of matching method

The common matching methods in the propensity score matching method include nearest neighbor matching, radius (caliper) matching, kernel matching, local linear regression matching, Markov matching, etc. Different matching methods lead to different matching estimators. There will also be bias in the matching estimator. Except in the “exact match” case, there is a *x*_*i*_ = *x*_*j*_ for all matches. The more common is non-exact matching, that is, only *x*_*i*_ ≈ *x*_*j*_ can be guaranteed. In the case of non-exact matching, the nearest neighbor one-to-one matching is to find a different group of nearest individuals for each individual to match. At this time, the deviation is smaller, but the variance is larger. One-to-many matching uses more information, which can reduce the variance, but the deviation increases. Abadie et al. (2004) believes that “one-to-four matching should be performed because the nearest neighbor one-to-four matching can minimize the mean square error in general.” This section selects nearest-neighbor one-to-one matching, nearest-neighbor one-to-four matching and radius (caliper) matching to test whether marital education matching affects household income inequality. Then kernel matching and Markov matching are used to test the robustness. When matching is performed, only individuals with overlapping propensity scores are usually retained to improve the quality of matching.

### 5.2. Data source and variable selection

The increase in the proportion of educationally homogeneous marriages may expand the per capita household income gap. This section uses the propensity score matching method (PSM) to verify the net impact of marriage education matching on household income inequality. To ensure the consistency and complementarity of empirical conclusions, this section continues to use the data from the 2013 China Household Income Survey (CHIP). The data processing method is also consistent; finally, 4,459 sample data are obtained. However, the explained variables of the propensity matching score method differ from those of the RIF method. The details are as follows.

(1) Explained variable: Degree of household income inequality

The main measures of household income inequality are income variance and the Gini coefficient. But this kind of coefficient can only reflect the overall degree of household income inequality. Even in the same region and era, the choice of marriage education matching pattern has different effects on household income. Generally speaking, the Gini coefficient represents the overall degree of household income inequality, ignoring individual heterogeneity. Therefore, this section uses the accurate income relative deprivation index to measure the degree of household income inequality. The relative deprivation index of income refers to “the higher the income level of a household in a specific group, the lower the relative deprivation it suffers, which is reflected in the reduction of income inequality.” Three commonly used relative deprivation indexes are the Yitzhaki index (Yitzhaki, 1979), the Kakwani index (Kakwani, 1984), and the podder index (Podder, 1996). The Kakwani index is dimensionless and has regularization characteristics. Taking the mean value of the Kakwani index is the Gini coefficient, and the relative deprivation within the group can be objectively measured (Ren and Pan, 2016). Based on this, this section selects the Kakwani index to calculate the relative deprivation index of household income. We can get households' per capita income deprivation through income measurement and ranking. The steps for measuring the Kakwani index are as follows.

If the per capita income of the sample households is arranged in ascending order, the household income distribution of the group is *Z* = (*z*_1_, *z*_2_, ⋯  , *z*_*n*_), and *z*_1_ ≤ *z*_2_ ≤ ⋯ ≤ *z*_*n*_. Then the relative income deprivation suffered by the *i* family *z*_*i*_ is calculated by the formula in Equation (16).


(16) $$\begin{array}{c} RD=\frac{1}{n\upsilon_{X}}\overset{n}{\underset{j=i+1}{\sum }}\left(z_{j}-z_{i}\right)=\chi_{z_{i}}^{+}\left[\left(\upsilon_{z_{i}}^{+}-z_{i}\right)/\upsilon_{Z}\right] \end{array}$$

Equation (16) $\upsilon_{z_{i}}^{+}$ is the sample mean value calculated by households in the sample *Z* whose household income exceeds *z*_*i*_. $\chi_{z_{i}}^{+}$ is the proportion of the household sample whose household income exceeds that of the total sample in the sample group *Z*. Then, we calculate the relative deprivation index of per capita household income. A negative relationship exists between household per capita income and household income deprivation—the lower the household per capita income in the sample, the more obvious the poverty.

Explanatory variable: whether it is an educationally homogeneous marriage (*edu*_*mar*).

When the spouse has the same educational background, it is an educationally homogeneous marriage with a value of 1; The value of non-educational homogeneous marriage is assigned to 0.

(3) The control variables are the same as above. The control variables are roughly divided into household head characteristics and household characteristics. The province where the household is located is a dummy variable. To verify the conclusion of the previous section, the spouse education gap (*edu*_*gap*) is added as the explanatory variable in this section. According to the number of years of education, academic qualifications are divided into four levels. The division of educational hierarchy has been described in detail above. In this paper, the four levels are assigned 1–4, respectively, and the absolute value of the spouse's education assignment subtraction is the spouse's education gap.

### 5.3. Analysis of empirical results

(1) Benchmark regression

In the first section, through the re-centralization influence function regression, it is concluded that the rise of the proportion of homogeneous marriage in education will expand the household income gap. The explanatory variable indexes of the RIF method and PSM method are different. Therefore, before using propensity score matching (PSM) to verify the net effect of marriage education matching, this section is tested again by composite panel OLS regression. And the regression analysis was carried out with the marriage education matching pattern and the spouse education gap as the explanatory variables, respectively. The results are shown in Table 4. The explanatory variable of the model (1) and model (2) is the matching pattern of marriage education. The explanatory variable of models (3) and model (4) is the educational gap between spouses. Models (1) and model (3) control the provincial effect.

**Table 4:** OLS regression results of the educational structure of marriage on the relative deprivation of family per capita income.

**Household income inequality**	**(1)**	**(2)**	**(3)**	**(4)**
Whether homogenous education marriage	0.0224^***^ (4.41)	0.0222^***^ (4.11)	—	—
Spouse education gap	—	—	−0.179^***^ (-4.57)	−0.177^***^ (-4.52)
Control variables	Yes	Yes	Yes	Yes
Fixed province effect	Yes	No	Yes	No
Sample size	4,459	4,459	4,459	4,459
Adj R-squared	0.1838	0.1756	0.1844	0.1762

The t-value is in parentheses.

***, ** and * represent the 1, 5, and 10% significance levels, respectively.

From Table 4, the positive matching of marriage education significantly affects the relative deprivation of household income. Model (1) shows that after the fixed province effect, the spouse's choice of educationally homogeneous marriage will increase the degree of household income deprivation by 0.0224 units, which is significant at the 1% confidence level. Model (3) shows that after the fixed province effect, when the spouse's education increase by one lever, the degree of deprivation of per capita household income will be reduced by 0.179 units. And it is significant at the 1% level. It also proves that the increase in the proportion of educationally homogenous marriages will increase household income inequality. In the model of no fixed province effect, education is still a positive choice to expand household income inequality. But the model without controlling the provincial impact is more obvious. The composite panel OLS regression with the relative deprivation index of household income as the explanatory variable obtains the same conclusion as the RIF regression. That is, the positive matching of marriage education will expand the household income gap. Benchmark regression analyzes the effect of educational homogeneity marriage. Next, this section uses propensity score matching (PSM) to verify the net impact of educational homogeneity marriage.

(2) Propensity score estimation and equilibrium test

This section takes “whether to choose educationally homogeneous marriage” as the intervention option to analyze the net effect of marriage education matching on household income inequality. First, we need to use the logit model to estimate the propensity score. The explained variable is binary: whether to choose homogeneous educational marriage. Explanatory variables are a series of observable covariates, such as household head and household characteristics. Table 5 shows the estimated results of propensity scores and matching effects.

**Table 5:** Estimated results of the Logit model (nearest neighbor matching method k = 1).

**Variable**	**Coefficient (Z value)**	**Standard error**	***P*-value**
age	−0.0331^**^ (-2.00)	0.0166	0.046
*age* ^2^	0.0005^***^ (3.09)	0.0001	0.002
*par*	−0.6963^***^ (-11.22)	0.062	0.000
*hea*	0.0554 (1.57)	0.0353	0.117
*job*	−0.6036^***^ (-4.76)	0.1268	0.000
*emp*	0.2038^***^ (3.9)	0.0522	0.000
*fam*	0.066^***^ (2.33)	0.0283	0.02
*age*_*gap*	−0.047^***^ (-5.07)	0.0093	0.000
Constant term	1.129^**^ (2.48)	0.4553	0.013

Fitting validity of Logit Model: LR chi2(11) = 205.18, Prob>chi2 = 0.0000, Pseudo R2 = 0.028, Log likelihood = −3,561.6171.

The t-value is in parentheses.

***, ** and * represent the 1, 5, and 10% significance levels, respectively.

Table 5 shows the characteristics of the household head, such as age, whether he has a job, and whether he is a member of the CPC; household characteristics, such as the number of husband and wife workers, household size, the age difference between husband and wife, all will affect the choice of marriage education mode. By summarizing these factors, we find that the younger the household head is, the more likely they are to choose an educationally homogeneous marriage. Groups with working heads of households are more likely to choose educationally heterogeneous marriage. Choosing an educationally homogeneous wedding is significantly influenced by personal characteristics and household background.

**Figure 4:**
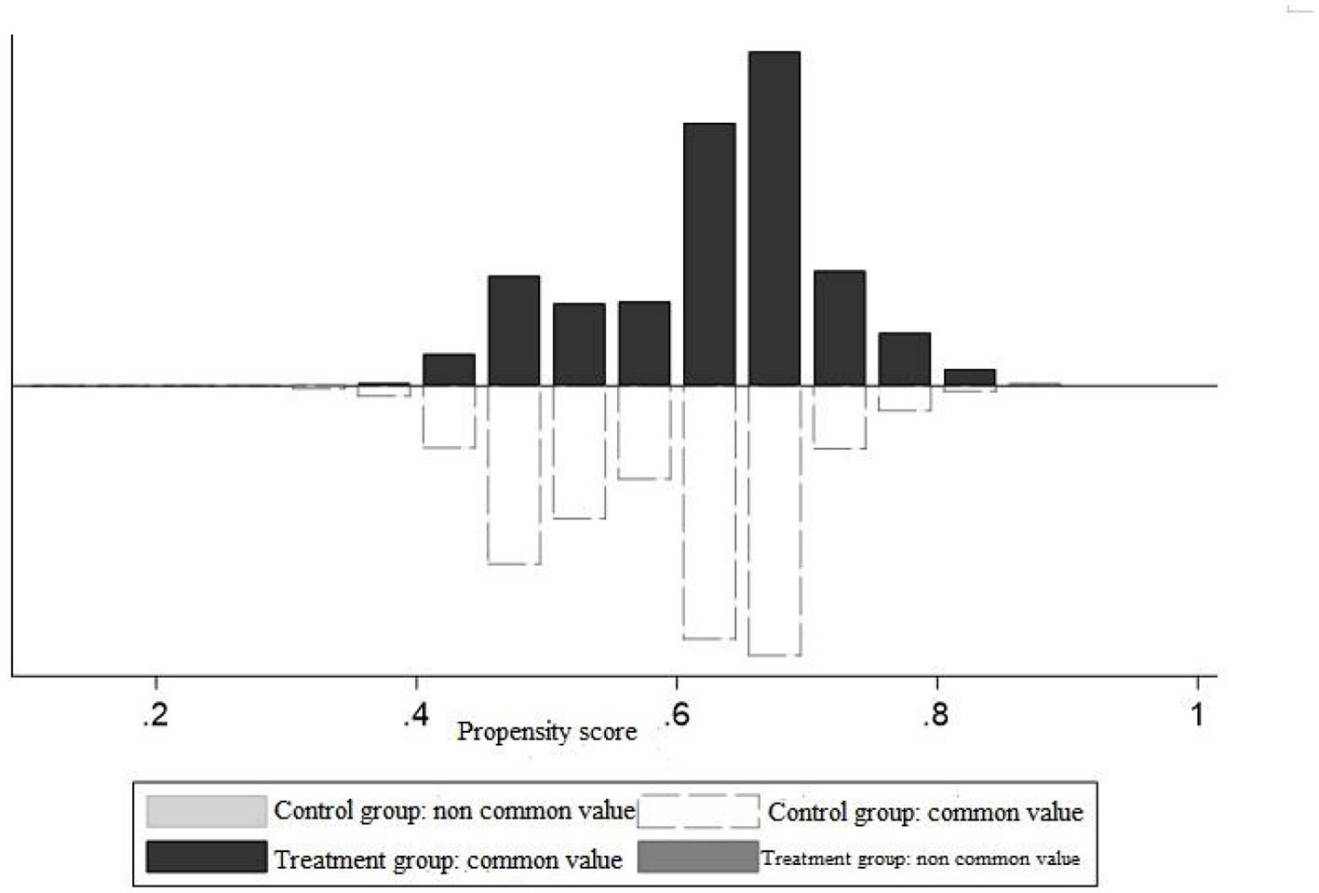
Common range of propensity scores.

It is necessary to check the balance and standard support before the propensity score matching (PSM) to ensure the reliability of the matching effect. Figure 4 shows the distribution of propensity scores in the experimental group and the control group. From the perspective of equilibrium results, almost all covariate standardization deviations have been reduced to a large extent after matching. The standard deviation of explanatory variables was all <10%, and the result of the *t*-test was not significant, which passed the joint test. It shows that after matching, there was no significant difference in the main characteristic variables between the experimental and control groups. The matching quality is relatively high and can satisfy the balance assumption. From Figure 4, essentially all observations fall within the expected value range of the propensity score, and only a small number of samples are lost in matching. After matching, the propensity score increased after the treatment of the experimental and control groups.

Similarly, the verification matching quality is relatively high. Then the propensity score is estimated. After the balance test, the propensity score matching can be performed next.

(3) Propensity score matching results

According to the above analysis, this section selects the nearest-neighbor one-to-one matching, nearest-neighbor one-to-four matching, and radius (caliper) matching to test the effect of marriage education matching on household income inequality. Table 6 shows these three methods' estimated results of the unmatched and matched propensity scores. Due to space limitations, only the results of ATT coefficients are listed in the table; ATU and ATE coefficients are no longer listed.

**Table 6:** Propensity score match results.

	**Match or not**	**Treatment group**	**Control group**	**ATT**	**Standard error**	**S.E**
Nearest-neighbor one-to-one matching	Unmatched	0.3563	0.3152	0.041^***^ (7.03)	0.008	0.0058
	Matching	0.356	0.3326	0.0235^***^ (3.01)		0.0078
Nearest-neighbor one-to-four matching	Unmatched	0.3562	0.3152	0.041^***^ (7.03)	0.0068	0.0058
	Matching	0.3560	0.329	0.027^***^ (4.11)		0.0066
Radius (caliper) matching	Unmatched	0.3563	0.3152	0.041^***^ (7.03)	0.0049	0.0058
	Matching	0.3553	0.332	0.0233^***^ (3.86)		0.006

In the nearest-neighbor matching setting, one-to-one matching is k = 1, and one-to-four matching is k = 4; the caliper matching range is set to 0.01. To avoid the effect of minor sample errors on the results, the above conclusion was estimated using a self-sampling iterative test to evaluate the effect's statistical significance and standard deviation. The number of iterations is set to 500.

The t-value is in parentheses.

***, ** and * represent the 1, 5, and 10% significance levels, respectively.

From the results in Table 6, choosing an educationally homogeneous marriage when it is not matched increases the degree of relative deprivation of household income. This coefficient is 0.041, which is higher than the coefficient of OLS regression above and is significant at the statistical level of 1%. After matching, according to the results of the average treatment effect given in Table 6, choosing educationally homogeneous marriage will still widen the household income gap. However, the influence coefficient decreases significantly compared to the unmatched condition. In the nearest neighbor one-to-one matching, the average treatment effect (ATT) of the relative deprivation degree of families who choose educationally homogeneous marriage is 0.0235, which was significant at the 1% level. This coefficient is 0.027 in the nearest-neighbor one-to-four matching, which is essential at the 1% level; This coefficient is 0.233 in radius matching, which is also significant at the 1% level. It is generally consistent with the OLS regression coefficient of 0.0224. After propensity score matching, marital education matching still increases the degree of relative deprivation of household income. However, this influence coefficient is much lower than when it is not matched. It also shows that the existence of omitted variables and the self-selectivity of marriage matching leads to the overestimation of the effect of marriage education matching on the degree of household income deprivation in the above empirical study.

The results of propensity score matching show that after correcting the possible selective bias, the net effect of choosing an educationally homogeneous marriage on the household's relative deprivation of per capita income is positive. Educational homogeneity marriage reduces the relative poverty of income of high-class households and increases the relative poverty of income of low-class households. Educational homogeneous marriage has become a means of class solidification, continuation, and maintenance. More marriageable men and women choose their spouses in their educational class groups. This paper uses propensity score matching to remove the solidification effect of “marriage within the class,” Choosing an educationally homogeneous marriage still widens the household income gap. The general employment ratio is roughly the same, and the policy does not adapt to China's current economic development level. The wages of technical talents trained in vocational schools are far lower than those of general skills taught in higher education.

## 6. Conclusion and policy

This study aims to demonstrate the effects of positive educational choices on family income inequality. Through the empirical test of the regression method of the re-centralized influence function and the propensity score matching method, it is finally concluded that the increase in the proportion of educational homogeneity in marriage will expand the degree of family income inequality. The specific conclusions are as follows:

(1) The increase in the proportion of homogeneous marriage in education produces the Matthew effect of “the richer the rich, the poorer the poor,” resulting in the expansion of household income inequality. The core of homogeneous educational marriage is the process of self-replicating and reproducing social stratum differentiation. If marriage matching is a random pattern, the random combination of marriageable men and women naturally does not affect the family income gap. Young people have few means to deal with uncertain risks, and marriage has become the best choice for young men and women to share risks in society.
(2) The propensity score matching (PSM) result shows that the net effect of choosing an educationally homogeneous marriage on the household's relative deprivation degree of per capita income is positive after correcting the possible selective deviation. The educational homogeneous marriage pattern reduces the relative deprivation of income of high-class households and increases the relative poverty of income of low-class households. After removing the solidification effect of “marriage within the class” by propensity score matching (PSM), choosing a homogeneous education marriage will still widen the household income gap. The actual inequality of educational opportunities is expanded into the inequality of family income through marriage education matching.
(3) The following conclusions were drawn from the analysis of the RIF regression method. Young men and women rely on marriage to ensure their quality of life and reduce risks, so they choose homogeneous educational marriage. The choice of educationally homogeneous marriage further widens the household income gap through the human capital level difference and intergenerational transmission caused by the spouse's education gap.

The main conclusion of this paper is that the increase in the proportion of homogeneous marriage in education has expanded household income inequality. But this is not the problem of educational homogeneity in marriage. Social progress and civilized development will inevitably make the marriage-matching mode a homozygous marriage-matching pattern. More individuals take “love” as the starting point of marriage and value the same values of potential spouses. If they have a sense of cultural identity, they need to search for a suitable spouse from the same life experience and similar value system. The essence of reducing the impact of marriage education matching on household income inequality is to reduce the correlation between marriage value and household income. Given the expansion of the household income gap caused by the positive choice of marriage matching in education, this paper puts forward three suggestions:

First of all, advocate the concept of pluralistic marriage. The traditional “Properly matched marriage” marriage mode ignores the individual's sense of happiness. The modern marriage mode should abandon the conventional marriage standard, take the individual's pursuit of happiness as the primary goal, advocate the concept of multiple marriages, and establish education on the correct concept of personal and family development.

Secondly, weaken the economic factors of positive choice of marriage. Gender discrimination in the labor market is diverse, and there is much hidden discrimination. The state needs to enact relevant laws and regulations to severely punish any discrimination by employers against female workers in childbearing and lactation periods and carry out various regulatory channels to protect women's rights and interests in the labor market. More importantly, we need to break the old ideas of the whole society, especially the prejudice against women in the labor market, let men participate in and share half of the family responsibilities, and realize the double equality between men and women in the labor market and family responsibilities.

Finally, we will promote skills training for family members with low and low education backgrounds to improve their total income. Currently, China cannot fully realize inclusive education, but it can enhance the income level of low-educated people through training and other ways. Providing practical and professional skills training can improve low-educated people's vocational competitiveness and increase their family income. At the same time, certain subsidies will be given to families with low incomes to promote children from families with low academic qualifications to receive higher education and prevent the family income gap from further expanding through intergenerational transmission.

The research of this paper also has some shortcomings, limited by the short investigation scope of the CHIP database and the lack of data in recent years. In addition, the rural household income gap has not been verified. Therefore, in future research, we will continue to look for more perfect data to study household income inequality. In addition, we cannot distinguish the return on education from other factors that affect income. The conclusion of this paper can not be used as a causal explanation for the positive choice of education between marriage matching and the household income gap. In the future, we will explore many ways to improve this conclusion.

## Data availability statement

Publicly available datasets were analyzed in this study. This data can be found at: http://ciid.bnu.edu.cn/.

## Author contributions

All authors listed have made a substantial, direct, and intellectual contribution to the work and approved it for publication.
